# Supplementary figures and images for: The effect of storage conditions on microbial communities in stool
Source: PLoS One. 2020 Jan 14;15(1):e0227486. doi: 10.1371/journal.pone.0227486 (PMC6959592; doi:10.1371/journal.pone.0227486)

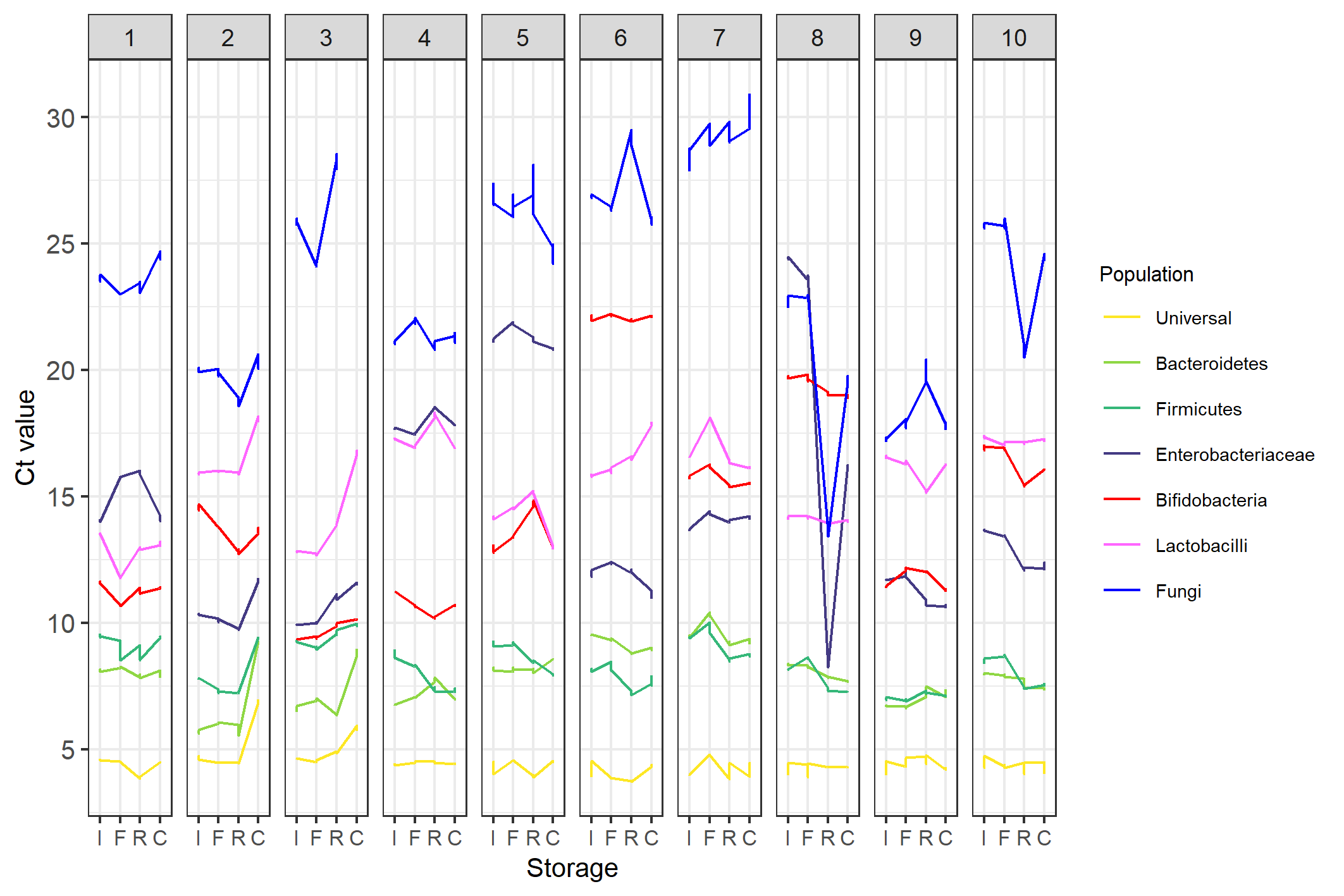

Supplement: S1 Fig — This plot shows the trend in abundance for each of the subjects (the ten boxes) at the different storage conditions. A lower Ct value represents a higher abundance. Ct = Cycle threshold; I = Immediate; F = Frozen; R = Room temperature; C = Cooler box. Samples 2C and 3C had a DNA input concentration < 30ng. (TIF) [file pone.0227486.s001.tif]
