# Supplementary material for: The effect of storage conditions on microbial communities in stool
Source: PLoS One. 2020 Jan 14;15(1):e0227486. doi: 10.1371/journal.pone.0227486 (PMC6959592; doi:10.1371/journal.pone.0227486)
Supplement: Attachment — Submitted filename: Response to reviewers_final.doc [file pone.0227486.s002.doc]

17 December 2019

**Response to Reviewers**

To the editor, Dr David Fredricks

We appreciate the comprehensive review of our research during the review process thus far. This letter serves as the summary of changes and/or rebuttal made by the authors in response to the reviewer comments below (indicated in italics). We aimed to address the issues raised during the review process in a comprehensive manner and adjusted the presentation of the results with additional statistical measurements to support our findings. We trust that this makes the findings more robust and clear to potential readers.

**Response to editor’s comments:**

*In addition, please explain how samples were homogenized, in detail.*

Samples were homogenized by manually mixing with the sterile spoon that was included in the stool collection tube. This has been indicated in the edited submission.

*We note that you included minors (age<18) in your study. Please provide additional details regarding minors' consent. In the ethics statement in the Methods and online submission information, please ensure that you have specified whether you obtained consent from parents or guardians.*

Informed consent was provided by parents/guardians. This has been indicated in the edited submission.

**Response to reviewer’s comments:**

*Line 55-57: This sentence should be revised: 1) microbial stability should not be limited to ‘specific’ populations, and should theoretically be consistent across populations, given the same conditions/storage/extraction/processing;*

*and 2) the data used by the authors are also ‘limited’ in both sample size (n=10) and population (children); therefore, the rationale in this sentence seems not justified.*

Microbiota and what influences them are diverse and unique in different settings; therefore, it is important to study the possible influences of study procedures in each setting. We have revised the sentence to reflect this.

*Line 76: The authors should state if subjects were on antibiotics at the time of collection*

- As this paper focuses on the differences related to storage relative to immediate extraction, we do not believe that it impacts the conclusions we have drawn.
- Participants were excluded if they had received ≥14 days of isoniazid or a fluroquinolone at enrolment, or if they had been treated for TB in the 12 months before. However, information regarding other antibiotics is not currently available to the authors as the trial is currently ongoing.
- Antibiotic usage data have been captured as part of the clinical trial, and have been requested from the trial data management team, but will only be made available for a larger sequencing-based study going forward.
- We have noted in the discussion section that differences in subjects, including the influence of antibiotics, will be investigated as part of a larger study going forward.

*Line 80-81: It is suggested that authors use a table or diagram to demonstrate the aliquoting of the samples and how many samples were compared within each collection method. They should also include which samples dropped out due to DNA yield/quality*

We have added a workflow (Fig 1) to demonstrate the aliquoting of samples. No samples were excluded from the qPCR analysis due to quality, due to the robustness of PCR. We have included a statement in the results section to this effect.

*Lines 125-132: Statistical analyses were insufficient to appropriately compare the stability of the microbial data between the storage methods. It is suggested that the authors use more quantitative comparisons, rather than basing their findings on the subjective figure interpretations. For example, the authors could compare the adjust mean Ct for each collected method, using linear mixed effects models, and calculate a p-value for each of the collection methods compared to the referent immediately-extracted group.*

Additional statistical measures have been described below and in the edited manuscript.

*Line 133: The random effect should be stated as the subject and not the sample (since there are multiple measurements on one subject)*

This has been revised in the manuscript.

*Line 126, 128, 143: Authors should avoid using terms as ‘cause’ and ‘effect’*

These terms have been revised in the manuscript where appropriate.

*Line 165-166: It is unclear what the authors are referring to*

This sentence referred to the fact that only a single qPCR reaction failed: The extracted sample from the cooler box group for subject 3 (3C) was not within the detection limit for the Fungal qPCR and was therefore excluded from qPCR analysis. We have revised this sentence in the edited manuscript to reflect our meaning more clearly.

*Figure 2: It is unclear what the 10 boxes represent. It also appears that many of the more abundant taxa were variable (not just the less abundant taxa)*

We have determined that Fig 3 represents our core findings most clearly and have kept it in the main text, but request to keep Fig 2 as a supplemental figure to show the variations for each subject (the ten boxes) across populations. The figure heading has been expanded to refer to the ten boxes as subjects.

*Figure 3: This seems redundant, and it is not clear that the subject cluster together. There needs to be a statistical test to quantify variation explained by subject versus storage*

We have added p-values to this figure to demonstrate that statistical tests support our findings. We have described the statistical tests that generated these values below, and in the manuscript in the methods section.

*Line 179: It is unclear why a single p-value is being used to compared multiple taxa abundances across the storage methods, this does not seem like the appropriate statistical test*

In consultation with the statisticians, it was felt that the population differences are inherently part of the influence and it was therefore included as a fixed effect in the linear mixed effect model. We used the entire data set, including all populations including the Universal amplification Cts (therefore, non-normalized Ct values) and indicated the p-value for differences related to storage as determined by the lme model. This model also output a p-value for population, and we have added that in the edited manuscript.

We agree with the reviewer that different taxa/populations may warrant separate statistical tests. For this reason, we decided to perform statistical testing using the normalized mean/median Ct values for each taxon. Instead of fitting linear mixed effect models for each storage group compared to the immediate as suggested by the reviewer, we performed Kruskal-Wallis multiple group comparisons. The reason for this is that linear mixed-effect models are more useful when more data is available – comparing the mean/median Ct values therefore diminishes the number of data points available.

Kind regards,

Kristien Nel Van Zyl
Division Medical Microbiology, Department of Pathology, Stellenbosch University
